# Helminth infections in slaughtered livestock of Qazvin Province, Iran: implications for food safety and public health

**DOI:** 10.1186/s13620-025-00325-z

**Published:** 2025-12-17

**Authors:** Fariba Najar Hoseini, Mohammadreza Mohammadi, Majid Pirestani, Armin Aligolzadeh, Leila Modarresnia, Mahendra Pal, Arash Zeinali, Aida Vafae Eslahi, Milad Badri

**Affiliations:** 1 Medical Microbiology Research Center, Qazvin University of Medical Sciences, Qazvin, Iran; 2 Department of Parasitology, Faculty of Medical Sciences, Tarbiat Modares University, Tehran, Iran; 3 Department of Pathobiology, Faculty of Veterinary Medicine, Urmia University, Urmia, Iran; 4 Narayan Consultancy of Veterinary Public Health, and Microbiology, B-103, Sapphire Lifestyle, Bharuch, Gujarat India; 5 Veterinary Head Office of Guilan Province, Rasht, Iran

**Keywords:** Helminths, Livestock, Food safety, One Health, Slaughterhouse, Iran

## Abstract

Helminthic parasites in livestock represent a significant challenge to both animal productivity and public health, particularly in developing countries. This study investigates the prevalence and diversity of helminth parasites in slaughtered livestock in Qazvin Province, north-western Iran an area with limited parasitological data.

A total of 6,885 slaughtered livestock including sheep (1,956), goats (1,322), and cattle (3,607) were examined between January 2024 and May 2025. Post-mortem inspections and coprological analyses were performed on major organs, with identification based on morphological and parasitological methods. Seasonal and species-specific patterns were analysed.

The overall prevalence of helminth infections was 47.10%, highest in sheep (75.35%) and goats (65.73%), and lowest in cattle (24.50%). Cystic echinococcosis (CE) was the most common cestode, with a prevalence of 11.32%, while *Dicrocoelium dendriticum* and *Fasciola hepatica* were the dominant trematodes. *Nematodirus spathiger* was the most prevalent nematode. Mixed infections were recorded in 9.59% of animals, particularly in sheep. Spring exhibited the highest seasonal prevalence across all host species.

The high burden and seasonal variation of helminth infections, especially zoonotic species like CE and *F. hepatica*, highlight critical food safety and public health risks. Enhanced meat inspection protocols, public education, and integrated control strategies are essential to reduce transmission and safeguard animal and human health in this region.

## Introduction

Parasites affecting livestock lead to diseases with major socioeconomic implications worldwide. The economic and agricultural damages they cause greatly impact the profitability of farms [1, 2]. Livestock helminthiases constitute a critical barrier to sustainable agriculture and public health security worldwide, particularly in developing economies where veterinary surveillance and control measures are often limited [3, 4]. Livestock such as cattle, sheep, and goats are commonly infected with a variety of helminthic parasites leading to substantial economic losses due to reduced meat and milk production, organ condemnation, and treatment costs [3, 5]. The public health implications are also noteworthy, as some helminths are zoonotic and may infect humans through consumption of undercooked meat or through contact with infected animal tissues.

Livestock can harbor a variety of helminth parasites that pose significant risks to human health. Among these, *Echinococcus granulosus* (the causative agent of cystic echinococcosis, CE), *Taenia solium*, *T. saginata*, *T. asiatica*, *Fasciola hepatica*, *F. gigantica*, and *Linguatula serrata* are of particular concern, as they can readily infect humans through the consumption of undercooked meat or offal, ingestion of contaminated water, or contact with contaminated environments. The presence of these parasites in slaughtered livestock highlights critical food safety challenges and underscores the need for effective public health interventions, including routine veterinary inspection, safe meat processing practices, and consumer education to reduce the risk of zoonotic transmission [6–9].

In endemic regions, these parasites perpetuate cycles of poverty and disease, underscoring the necessity of One Health approaches that integrate veterinary, medical, and environmental interventions. In Iran, ruminant husbandry is a major component of rural livelihoods and national food supply, making the surveillance of parasitic infections a priority for both veterinary and public health sectors. Although various studies have documented the presence of helminths in Iranian livestock, region-specific and large-scale data remain scarce, especially regarding mixed infections and species-specific prevalence.

Livestock production contributes approximately 40% to the total global agricultural gross value. It is also the largest user of land worldwide, both directly through grazing and indirectly through the cultivation of fodder and feed grains [10]. The rapid growth of the human population, coupled with increasing food demands, has driven a swift transformation in livestock production systems [11]. The wide scale of global livestock systems means that changes in animal health particularly regarding parasite prevalence can have far-reaching impacts beyond individual farms and across multiple sectors [12].

Traditional animal husbandry remains a widespread source of livelihood among rural communities in Iran. Qazvin Province, located in the north-western region of the country, serves as a major center for livestock farming [13, 14]. Although numerous studies in Iran have investigated parasitic infections in livestock, data specific to this region remain limited. Therefore, the present study was conducted in selected slaughterhouses across Qazvin Province, Iran, encompassing a range of facilities, processing methods, and management practices.

This study aimed to determine the prevalence and diversity of helminth parasites in slaughtered sheep, goats, and cattle in Qazvin Province of Iran. By identifying the species involved and their infection rates, the findings contribute to a better understanding of the parasitic burden in Iranian livestock and offer insight into potential zoonotic risks and necessary control strategies. Moreover, it outlines the need to upgrade meat inspection services, health literacy among the community, and integrated parasite control essential steps towards animal welfare, food safety, and public health protection in resource-constrained settings.

## Materials and methods

### Study area and sampling

**Fig. 1:**
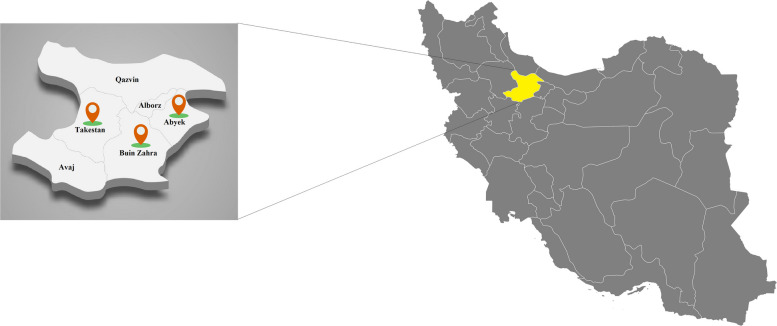
Geographical Location of Qazvin Province in Northwestern Iran

The current cross-sectional study was carried out between January 2024 and May 2025 in Qazvin Province, located in north-western Iran (Fig. 1). The province includes six counties: Abyek, Avaj, Alborz, Buinzahra, Takestan, and Qazvin. The region experiences an average annual rainfall of approximately 280 mm, with precipitation levels declining from north to south. The mean temperature is 15.5°C, and the estimated potential evapotranspiration is around 2,200 mm [15].

A total of 6,885 slaughtered livestock, including 3,607 cattle, 1,956 sheep, and 1,322 goats were examined for helminth infections in Qazvin province, north-western Iran. Animals were selected using a simple random sampling approach during routine meat inspections under the supervision of veterinary authorities (Table 1).

**Table 1:** The number and prevalence of helminthic parasites found in slaughtered ruminants

Helminth		Animals (*n* = 6,885)			No. positive (%)	95% CI^*^
		**Sheep (*n* = 1,956)**	**Goats (*n* = 1,322)**	**Cattle (*n* = 3,607)**		
**Class**	**Species/Larval stage**	**No. positive (%)**	**No. positive (%)**	**No. positive (%)**		
Trematoda	*Dicrocoelium dendriticum*	181 (9.25)	63 (4.76)	175 (4.85)	419 (6.11)	6.11 (5.60–6.70)
	*Fasciola hepatica*	126 (6.44)	45 (3.40)	137 (3.79)	308 (4.49)	4.49 (4.00–5.00)
	*Fasciola gigantica*	62 (3.16)	22 (1.66)	78 (2.16)	162 (2.36)	2.36 (2.00- 2.80)
	*Paramphistomum cervi*	57 (2.91)	21 (1.58)	42 (1.16)	120 (1.75)	1.75 (1.50–2.10)
Cestoda	Cystic echinococcosis	268 (13.70)	197 (14.90)	311 (8.62)	776 (11.32)	11.32 (11.00–12.00)
	*Moniezia benedeni*	105 (5.36)	43 (3.25)	11 (0.30)	159 (2.32)	2.32 (2.00–2.70)
	*Moniezia expansa*	149 (7.61)	195 (14.75)	-	344 (5.02)	5.02 (4.50–5.60)
	*Cysticercus bovis*	-	-	18 (0.49)	18 (0.26)	0.26 (0.10–0.40)
	*Cysticercus tenuicollis*	123 (6.28)	101 (7.63)	-	224 (3.27)	3.27 (2.90–3.70)
	*Stilesia globipunctata*	21 (1.07)	6 (0.45)	-	27 (0.39)	0.39 (0.20–0.50)
Nematoda	*Marshallagia marshalli*	93 (4.75)	56 (4.23)	-	149 (2.17)	2.17 (1.90–2.50)
	*Haemonchus contortus*	87 (4.44)	27 (2.04)	-	114 (1.66)	1.66 (1.40–2.00)
	*Nematodirus spathiger*	113 (5.77)	62 (4.68)	-	175 (2.55)	2.55 (0.22–3.00)
Pentastomida	*Linguatula serata*	89 (4.55)	31 (2.34)	112 (3.10)	232 (3.38)	3.38 (3.00–3.80)
**Total**		1,474 (75.35)	869 (65.73)	884 (24.50)	3,227 (47.10)	47.10 (46.00–48.00)

^*^Confidence Interval 95% (95% CIs) calculate via a website (https://www.statskingdom.com/proportion-confidence-interval-calculator.html)

### Parasitological examination

Post-mortem examinations were carried out on major organs including the liver, lungs, kidneys, intestines, rumen, abomasum, mesentery, heart, brain, and lymph nodes.

Collected samples, both visibly infected and those suspected of parasitic involvement, were preserved in ice-cooled containers and promptly transferred to the laboratory. Upon arrival, the organs were dissected and inspected for helminth parasites. Cystic lesions were dissected and examined microscopically to identify larval stages of cestodes such as *Cysticercus* and CE. Gastrointestinal tracts were incised, and their contents were washed and filtered for helminth recovery.

In addition, a coprological examination was performed according to standardized protocols. To ensure the accuracy and reliability of the analysis, quality control measures were implemented throughout the sample preparation and evaluation process. Approximately 3 g of fecal material were mixed with 42 mL of 0.1% sodium hydroxide (NaOH) solution and thoroughly stirred. The mixture was then filtered, and sedimentation was allowed for 5 min to isolate parasite eggs. This sedimentation step was repeated until most fecal debris was removed. The resulting sediment was resuspended in 5 mL of tap water containing a drop of methylene blue to facilitate staining. The prepared samples were transferred to petri dishes and examined under low-power magnification. Parasite eggs were identified and counted by systematically scanning the entire dish. Adult helminths were preserved in 70% ethanol and later identified morphologically using taxonomic keys [5].

Both post-mortem and coprological analyses were performed on each animal. Coprological analysis was primarily employed to supplement post-mortem findings, allowing for the identification of intestinal helminths that might not be visible on gross inspection. Positive cases were noted if parasites or eggs were recovered by either test, ensuring comprehensive detection and reducing false negatives.

### Data analysis

Prevalence was calculated as the number of infected animals divided by the total number of animals examined for each species and helminth. Confidence intervals (95%) were calculated using the Wilson score interval method, implemented in the StatsKingdom online proportion confidence interval calculator (https://www.statskingdom.com/proportion-confidence-interval-calculator.html).

Visualizations, including clustered bar charts and heatmaps, were created using Python (v3.x) with the Seaborn and Matplotlib packages to illustrate seasonal patterns of parasite prevalence [16, 17].

### Molecular analysis

To complement morphological identification and confirm the taxonomic status of selected helminths, molecular analysis was performed on representative isolates. Approximately 25 mg of adult worms or larval cyst tissues were homogenized and lysed in the provided buffer containing Proteinase K, followed by incubation at 60 °C for 1 h. DNA was purified using a commercial tissue DNA extraction kit (Favorgen, Taiwan) according to the manufacturer’s protocol, eluted in 100 µL of elution buffer, and stored at − 20 °C until use.

The quality and quantity of extracted DNA were verified by 1% agarose gel electrophoresis and spectrophotometric analysis using a NanoDrop™ 2000 (Thermo Scientific, USA). Only samples with intact high-molecular-weight DNA and an A260/A280 ratio between 1.8 and 2.0 were used for PCR amplification.

The internal transcribed spacer (ITS) region of the nuclear ribosomal DNA, a widely used marker for species-level identification and phylogenetic analysis of helminths [18, 19], was amplified using the universal primers BD1 (5′-GCTGTAACAAGGTTTCCGTA-3′) and BD2 (5′-TATGCTTAAATTCAGCGGGT-3′).

PCR amplification was carried out in a 25 µL reaction mixture containing 12.5 µL of 2X PCR Master Mix (Ampliqon, Denmark), 1 µL of each primer (10 pmol), 2 µL of template DNA, and 8.5 µL of nuclease-free water. The thermal cycling conditions included an initial denaturation at 94 °C for 5 min, followed by 35 cycles of denaturation at 94 °C for 30 s, annealing at 55 °C for 45 s, and extension at 72 °C for 1 min, with a final extension at 72 °C for 7 min. The expected size of the amplified ITS fragment was approximately 1,000–1,300 bp depending on the helminth species.

PCR products were verified by electrophoresis on 1.5% agarose gels stained with DNA safe stain and visualized under UV transillumination. Selected amplicons were purified and subjected to bidirectional Sanger sequencing (Bioneer, South Korea). The resulting chromatograms were examined, trimmed, and assembled using BioEdit version 7.2.6 and Chromas 2.6.6 to ensure sequence accuracy. Only high-quality bidirectional reads without ambiguous base calls were used for downstream analyses.

The obtained sequences were compared with reference sequences in GenBank using BLASTn (https://blast.ncbi.nlm.nih.gov). Species assignment was based on ≥ 98% nucleotide identity and ≥ 95% query coverage. Multiple sequence alignments were performed using the MUSCLE algorithm implemented in MEGA version 11 prior to phylogenetic reconstruction.

For phylogenetic analysis, separate Maximum Likelihood trees were generated for nematodes, cestodes, and trematodes based on ITS sequences. The best-fitting nucleotide substitution models were selected according to the lowest Bayesian Information Criterion (BIC) values: the General Time Reversible (GTR) model for nematodes and trematodes, and the Hasegawa–Kishino–Yano (HKY) model for cestodes. In all phylogenetic trees, no external outgroup was included, as the objective of this study was to confirm species identity and assess genetic relatedness among regional isolates rather than to infer deep evolutionary relationships. Therefore, all trees were midpoint-rooted to provide a balanced visualization of intra-group clustering and to minimize topological distortion that might result from distantly related taxa. Bootstrap analyses with 1,000 replicates were performed to evaluate the robustness of the inferred clades [20].

## Results

### Morphological identification

**Fig. 2:**
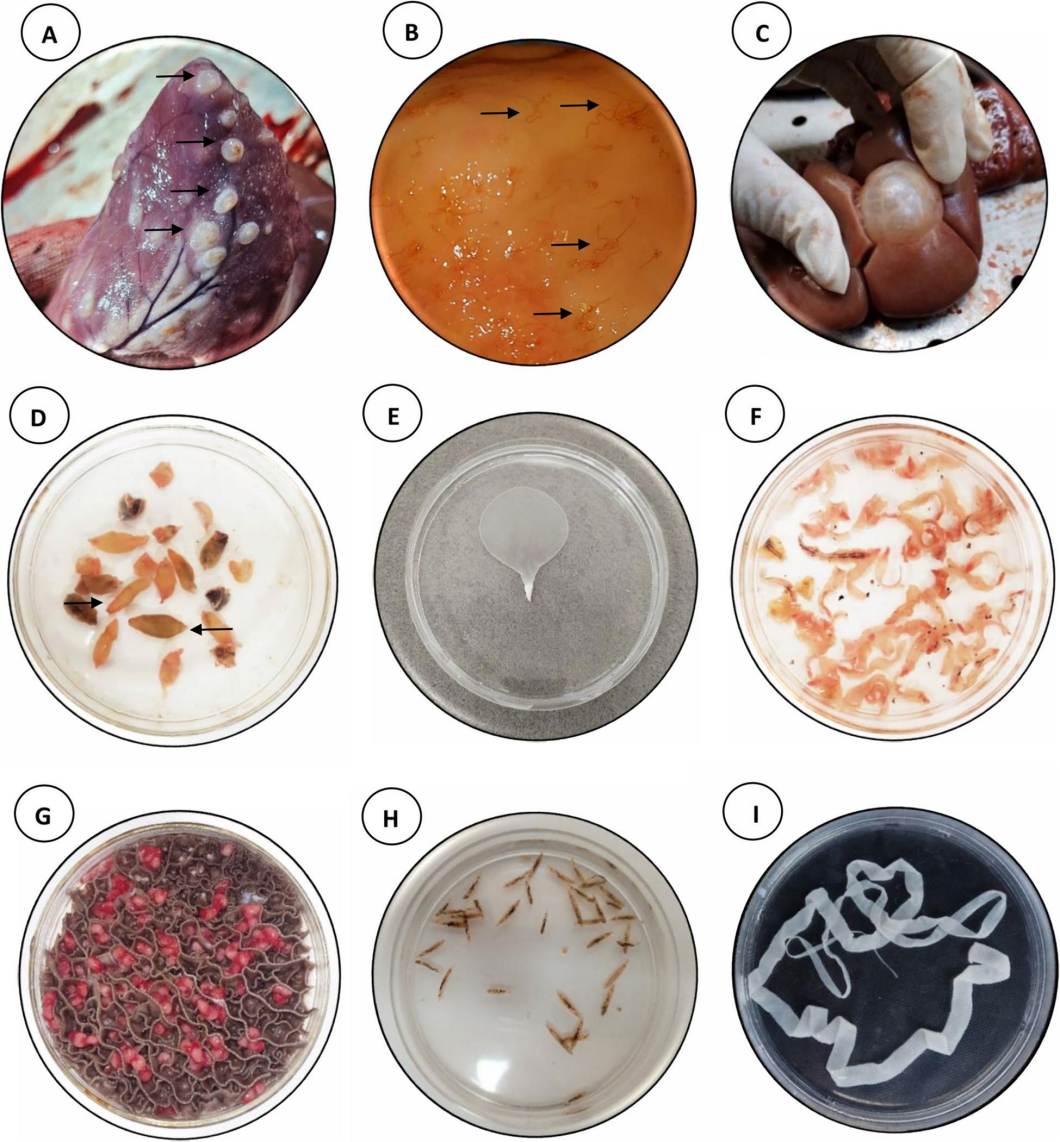
*Cysticercus bovis* in the heart muscle of cattle (**A**), *Marshallagia marshalli* in the abomasum of sheep (**B**), Cystic echinococcosis in the kidney of cattle (**C**), *Fasciola hepatica* (right arrow) and *F. gigantica* (left arrow) in goat (**D**),*Cysticercus tenuicollis* (**E**), *F. gigantica* in sheep (**F**), Adult of *Paramphistomum cervi* in the rumen of cattle (**G**), *Dicrocoelium dendriticum* (**H**), and *Moniezia benedeni* (**I**)

**Fig. 3:**
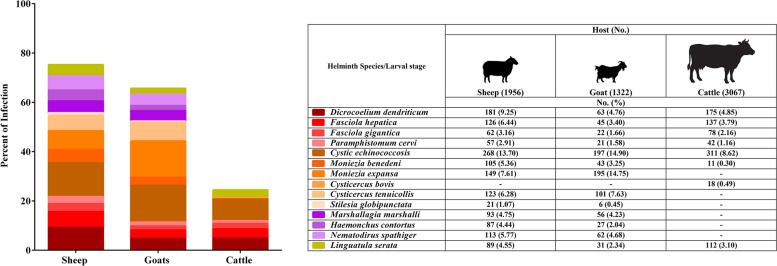
The bar chart showing the distribution of helminth parasites in slaughtered livestock in Qazvin Province

Out of all examined livestock, the overall prevalence of helminth infections was 47.10% (95% CI: 46.00–48.00). Species-specific prevalence rates were highest in sheep at 75.35%, followed by goats at 65.73%, and cattle at 24.50% (Fig. 2 & 3, Table 1).

In sheep, the most common trematode was *Dicrocoelium dendriticum* (9.25%), followed by *Fasciola hepatica* (6.44%), *F. gigantica* (3.16%), and *Paramphistomum cervi* (2.91%). Among cestodes, CE was the most prevalent (13.70%), followed by *Moniezia expansa* (7.61%), *Cysticercus tenuicollis* (6.28%), *M. benedeni* (5.36%), and *Stilesia globipunctata* (1.07%). The most frequently observed nematodes in sheep included *Nematodirus spathiger* (5.77%), *Marshallagia marshalli* (4.75%), and *Haemonchus contortus* (4.44%). In addition, *Linguatula serrata* was found in 4.55% of sheep (Fig. 2 & 3).

Goats showed a total helminth prevalence of 65.73%. Among trematodes, *D. dendriticum* (4.76%) was most prevalent, followed by *F. hepatica* (3.40%), *F. gigantica* (1.66%), and *P. cervi* (1.58%). Cestodes were also commonly detected, with *M. expansa* (14.75%) and CE (14.90%) having the highest prevalence, followed by *C. tenuicollis* (7.63%), *M. benedeni* (3.25%), and *S. globipunctata* (0.45%). Among nematodes, *N. spathiger* (4.68%) was most frequent, followed by *M. marshalli* (4.23%) and *H. contortus* (2.04%). *L. serrata* was detected in 2.34% of goats (Fig. 2 & 3).

Cattle showed the lowest overall infection rate at 24.50%. The predominant trematode was *D. dendriticum* (4.85%), followed by *F. hepatica* (3.79%), *F. gigantica* (2.16%), and *P. cervi* (1.16%). CE was the most prevalent cestode in cattle (8.62%), while *M. benedeni* and *C. bovis* were found at 0.30% and 0.49%, respectively. Other cestodes such as *M. expansa*, *C. tenuicollis*, and *S. globipunctata* were not detected. No nematodes were observed in cattle; however, *L. serrata* was recorded in 3.10% of the cattle examined (Fig. 2 & 3, Table 1).

### Mixed infections

Out of 6,885 slaughtered livestock examined, mixed infections were recorded in 657 animals (9.59%). Sheep exhibited the highest proportion of mixed infections, with 317 cases (16.20%), followed by goats with 166 cases (12.55%), and cattle with 174 cases (4.82%) (Table 2).

**Table 2:** Mixed infections in 6,885 slaughtered ruminants examined in Qazvin Province, Iran between January 2024 and May

Animals	Mixed infection (No. animals)
Sheep	Cystic echinococcosis + *Cysticercus tenuicollis* + *Marshallagia marshalli* (28) *Dicrocoelium dendriticum* + Cystic echinococcosis + *Haemonchus contortus* (16) *Moniezia benedeni* + *Fasciola gigantica* + *Linguatula serata* (11) *Nematodirus spathiger* + Cystic echinococcosis (45) *Stilesia globipunctata* + Cystic echinococcosis + *Fasciola hepatica* (13) *Cysticercus tenuicollis* + *Dicrocoelium dendriticum* + *Marshallagia marshalli* (4) Cystic echinococcosis + *Paramphistomum cervi* + *Fasciola gigantica* (6) *Haemonchus contortus* + *Moniezia benedeni* + *Dicrocoelium dendriticum* (15) Cystic echinococcosis + *Cysticercus tenuicollis* (52) *Moniezia benedeni* + *Fasciola hepatica* + *Linguatula serata* + *Cysticercus tenuicollis* (18) *Moniezia expansa* + Cystic echinococcosis (26) *Nematodirus spathiger* + *Dicrocoelium dendriticum* (21) *Marshallagia marshalli* + *Paramphistomum cervi* + Cystic echinococcosis (8) *Moniezia expansa* + *Fasciola hepatica* (42) *Moniezia benedeni* + *Dicrocoelium dendriticum* + *Haemonchus contortus* (12)
Total (%)	317 (16.20)
Goats	Cystic echinococcosis + *Moniezia expansa* + *Dicrocoelium dendriticum* (24) *Moniezia expansa* + *Haemonchus contortus* + *Cysticercus tenuicollis* (11) *Moniezia benedeni* + *Linguatula serata* + Cystic echinococcosis + *Cysticercus tenuicollis* (4) *Paramphistomum cervi* + *Fasciola gigantica* + *Cysticercus tenuicollis* (7) *Fasciola gigantica* + *Moniezia expansa* + *Cysticercus tenuicollis* (3) *Paramphistomum cervi* + *Moniezia benedeni* + Cystic echinococcosis + *Marshallagia marshalli* (10) Cystic echinococcosis + *Cysticercus tenuicollis* (32) Cystic echinococcosis + *Moniezia expansa* (41) *Moniezia benedeni* + *Dicrocoelium dendriticum* + Cystic echinococcosis (2) *Marshallagia marshalli* + *Moniezia expansa* + *Cysticercus tenuicollis* (6) *Fasciola hepatica* + *Nematodirus spathiger* + *Moniezia expansa* (6) *Dicrocoelium dendriticum* + *Stilesia globipunctata* (3) *Moniezia expansa* + *Stilesia globipunctata* (1) *Moniezia expansa* + Cystic echinococcosis + *Dicrocoelium dendriticum* (13) *Fasciola hepatica* + *Marshallagia marshalli* (3)
Total (%)	166 (12.55)
Cattle	*Dicrocoelium dendriticum* + Cystic echinococcosis (32) Cystic echinococcosis + *Dicrocoelium dendriticum* + *Linguatula serata* (16) *Dicrocoelium dendriticum* + *Fasciola hepatica* (21) Cystic echinococcosis + *Linguatula serata* + *Paramphistomum cervi* (11) *Dicrocoelium dendriticum* + *Cysticercus bovis* + *Linguatula serata* (7) *Fasciola gigantica* + *Paramphistomum cervi* (23) *Moniezia benedeni* + *Cysticercus bovis* (3) *Fasciola gigantica* + *Moniezia benedeni* (7) Cystic echinococcosis + *Fasciola hepatica* (25) *Linguatula serata* + Cystic echinococcosis (29)
Total (%)	174 (4.82)

2025

In sheep, the most frequent mixed infection was CE and *C. tenuicollis*, observed in 52 animals, followed by *N. spathiger* and CE (45 cases), and *M. expansa* alongside *F. hepatica* (42 cases).

Among goats, the most common mixed infections were CE and *M. expansa* in 41 cases and CE alongside *C. tenuicollis* in 32 cases (Table 2).

In cattle, mixed infections were generally less prevalent. The most frequent combination was *D. dendriticum* and CE (32 cases), followed by *L. serrata* and CE observed in 29 cases (Table 2).

### Temporal patterns and host-specific trends in helminth prevalence

Across all host species, helminth infections were most common during spring, gradually declining toward winter. Spring accounted for the highest number of positive cases in sheep (492), goats (352), and cattle (347), while winter showed the lowest infection rates across all host species.

Among sheep, the most frequently detected parasite was CE (13.70%), with the highest number of cases reported in spring (98 cases) and the lowest in winter (50 cases) (Table 3).

**Table 3:** Seasonal distribution and characterization of helminthic parasites in ruminants

Parasite	Season				Total (%)
	**Spring**	**Summer**	**Autumn**	**Winter**	
	**No. positive**				
	**Sheep (*n* = 1,956)**				
*Dicrocoelium dendriticum*	58	61	37	25	181 (9.25)
*Fasciola hepatica*	49	24	41	12	126 (6.44)
*Fasciola gigantica*	19	22	11	10	62 (3.16)
*Paramphistomum cervi*	13	27	17	-	57 (2.91)
Cystic echinococcosis	98	55	65	50	268 (13.70)
*Moniezia expansa*	44	61	32	12	149 (7.61)
*Moniezia benedeni*	28	59	13	5	105 (5.36)
*Cysticercus tenuicollis*	46	33	12	32	123 (6.28)
*Stilesia globipunctata*	5	16	-	-	21 (1.07)
*Haemonchus contortus*	38	18	31	-	87 (4.44)
*Nematodirus spathiger*	31	42	29	11	113 (5.77)
*Marshallagia marshalli*	48	24	-	21	93 (4.75)
*Linguatula serata*	15	22	35	17	89 (4.55)
Total (%)	492 (25.15)	464 (23.72)	323 (16.51)	195 (9.96)	1,474 (75.35)
	**Goats (*n* = 1,322)**				
*Dicrocoelium dendriticum*	25	19	12	7	63 (4.76)
*Fasciola hepatica*	16	21	-	8	45 (3.40)
*Fasciola gigantica*	11	6	3	2	22 (1.66)
*Paramphistomum cervi*	8	12	-	1	21 (1.58)
Cystic echinococcosis	94	52	46	5	197 (14.90)
*Moniezia expansa*	59	81	55	-	195 (14.75)
*Moniezia benedeni*	19	16	2	6	43 (3.25)
*Cysticercus tenuicollis*	47	25	-	29	101 (7.63)
*Stilesia globipunctata*	-	5	-	1	6 (0.45)
*Haemonchus contortus*	15	4	8	-	27 (2.04)
*Nematodirus spathiger*	31	22	7	2	62 (4.68)
*Marshallagia marshalli*	18	23	9	6	56 (4.23)
*Linguatula serata*	9	18	4	-	31 (2.34)
Total (%)	352 (26.62)	304 (22.99)	146 (11.04)	67 (5.06)	869 (65.73)
	**Cattle (*n* = 3,607)**				
*Dicrocoelium dendriticum*	66	47	43	19	175 (4.85)
*Fasciola hepatica*	41	58	30	8	137 (3.79)
*Fasciola gigantica*	23	42	13	-	78 (2.16)
*Paramphistomum cervi*	26	11	5	-	42 (1.16)
Cystic echinococcosis	102	73	84	52	311 (8.62)
*Moniezia benedeni*	8	3	-	-	11 (0.30)
*Cysticercus bovis*	4	5	-	9	18 (0.49)
*Linguatula serata*	77	13	8	14	112 (3.10)
Total (%)	347 (9.62)	252 (6.98)	183 (5.07)	102 (2.82)	884 (24.50)

*Dicrocoelium dendriticum* (9.25%) and *M. expansa* (7.61%) were also common, peaking in summer. *Fasciola hepatica* was detected throughout the year, with the highest occurrence in Spring (49 cases) and the lowest in winter (12 cases). Seasonal trends showed the highest overall infection burden in spring (492 positive cases), gradually decreasing through summer (464), autumn (323), and winter (195) (Table 3).

In goats, CE had the highest prevalence (14.90%), with the majority of cases recorded in spring (94 cases). *Moniezia expansa* (14.75%) and *C. tenuicollis* (7.63%) were also prominent. Notably, *F. hepatica* was absent in autumn, while *F. gigantica* showed a low and declining trend across all seasons. Overall helminth infections in goats were most prevalent in spring (352 positive cases), followed by summer (304), autumn (146), and winter (67) (Table 3).

In cattle, CE was again the most commonly identified helminth (8.62%), with a marked peak in spring (102 cases). *Dicrocoelium dendriticum* (4.85%) and *F. hepatica* (3.79%) were also frequently detected. *Linguatula serrata* (3.10%) showed an unusual trend, with the highest incidence in spring (77 cases) and another peak in winter (14 cases). Helminth infections in cattle followed a similar seasonal trend, being most prevalent in spring (347 cases) and least in winter (102 cases) (Table 3).

**Fig. 4:**
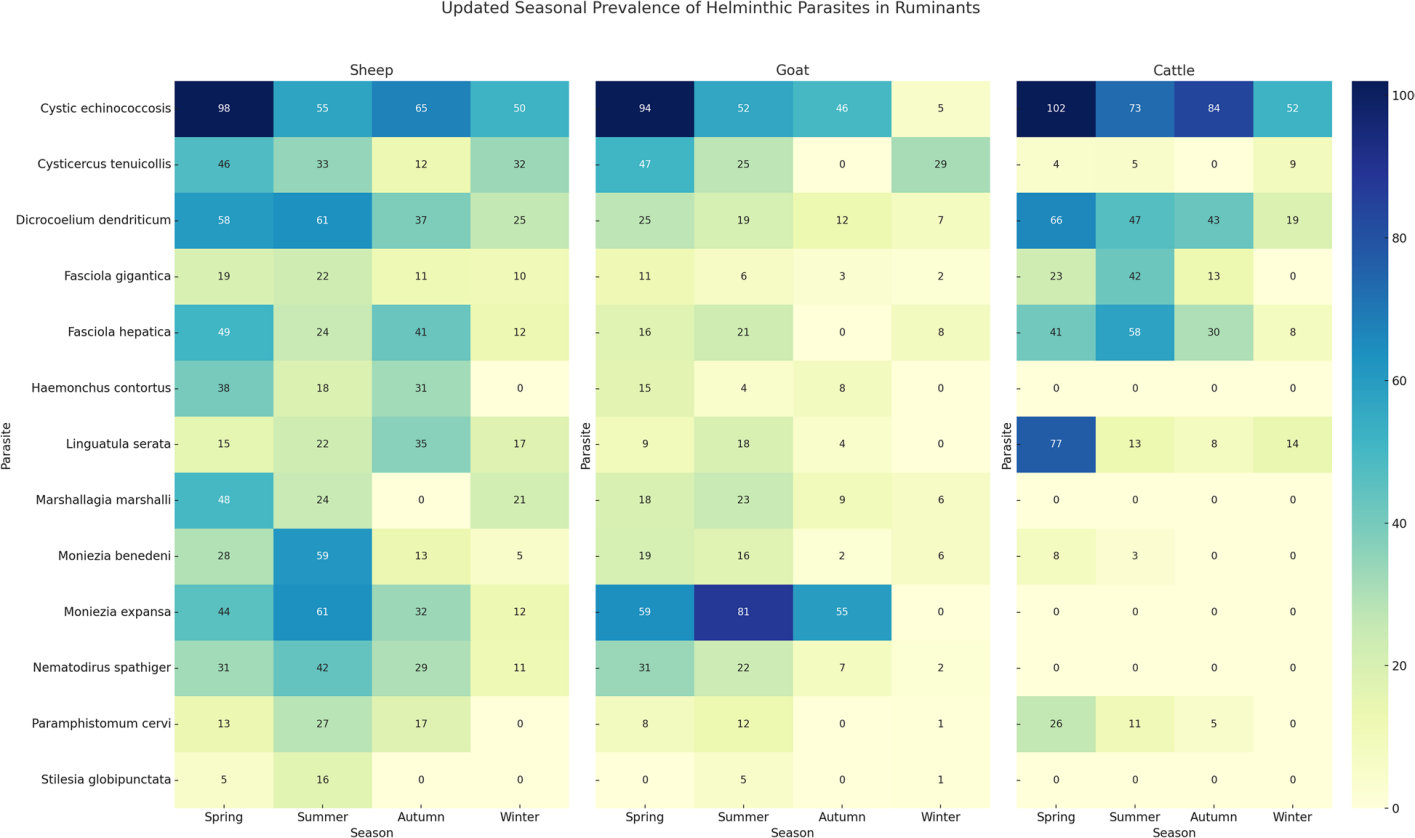
The seasonal heatmaps illustrate distinct patterns in the prevalence of helminthic parasites among sheep, goats, and cattle across the four seasons

The seasonal heatmaps illustrate distinct patterns in the prevalence of helminthic parasites among sheep, goats, and cattle across the four seasons (Fig. 4).

In sheep, the highest parasite burdens were observed in spring and summer, especially for CE (98 and 55 cases), *M. expansa* (44 and 61), and *M. benedeni* (28 and 59). Notably, *F. hepatica* was most common in spring (49) and autumn (41), while *H. contortus* was almost exclusively detected in spring and autumn, with no cases reported in winter. Several parasites such as *P. cervi* and *S. globipunctata* were not detected during winter, indicating seasonal transmission patterns (Fig. 4).

Among goats, similar seasonal trends were observed, with the highest prevalence again occurring in spring and summer. *Moniezia expansa* showed particularly high counts (59 in spring, 81 in summer), and CE remained relatively frequent. However, many parasites such as *F. hepatica*, *P. cervi*, and *C. tenuicollis* showed a sharp drop or were absent during autumn and winter, suggesting seasonal interruption in their life cycles or lower host exposure (Fig. 4).

In cattle, while the parasite load was generally lower compared to sheep and goats, CE showed consistent prevalence throughout all seasons, with a peak in spring (102 cases). *Fasciola hepatica* was more prevalent in summer, and *L. serrata* maintained presence across all seasons, especially spring (77) and winter (14), suggesting differences in exposure, management, or host susceptibility (Fig. 4).

### Molecular identification

**Fig. 5:**
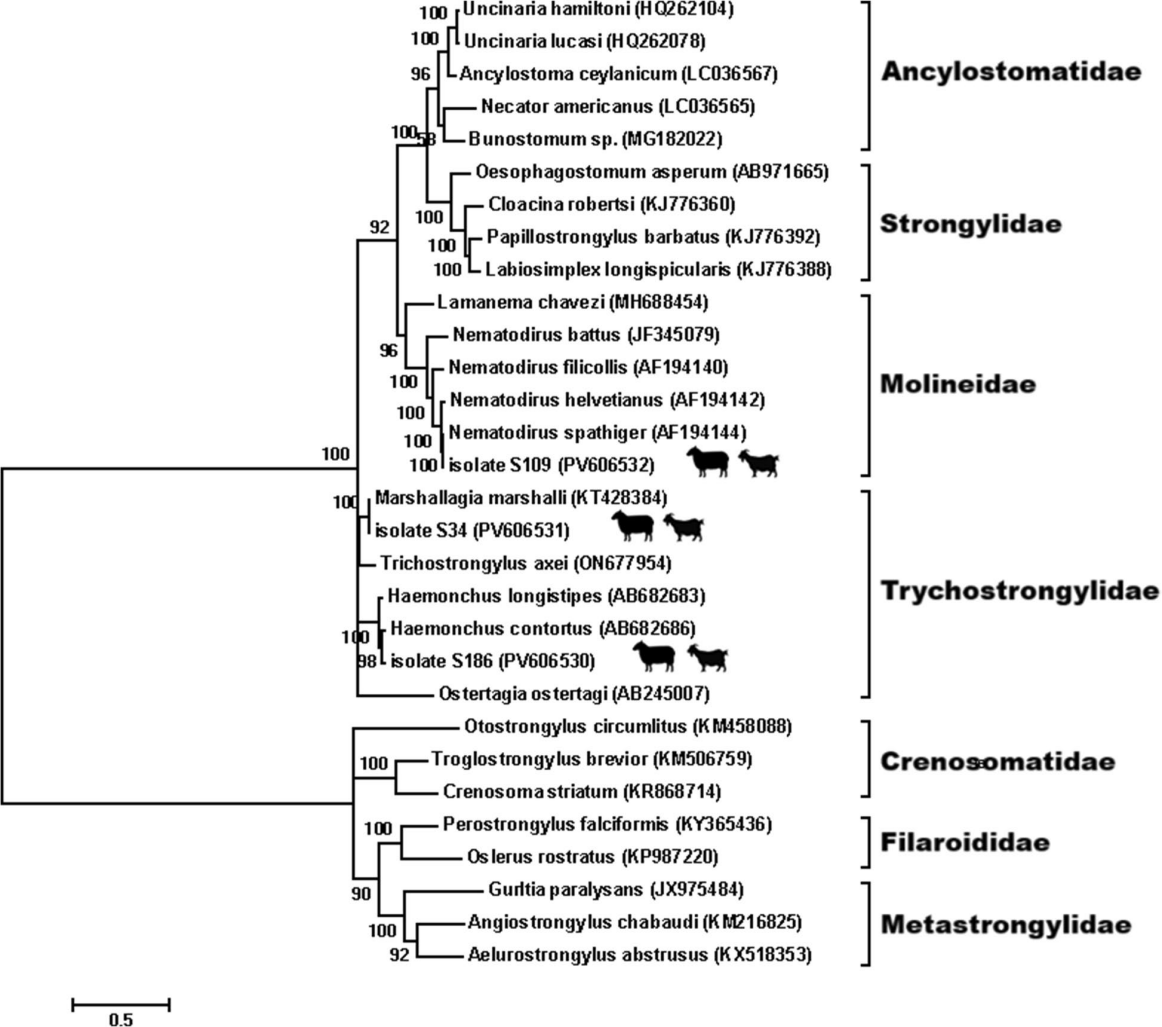
Evolutionary analysis by the Maximum Likelihood method of nematodes based on ITS region. The phylogeny was inferred using the Maximum Likelihood method and General Time Reversible model of nucleotide substitutions and the tree with the highest log likelihood (-19,332.54) is shown. The percentage of replicate trees in which the associated taxa clustered together (1000 replicates) is shown next to the branches

Three nematode species were confirmed molecularly: *H. contortus* (PV606530), *M. marshalli* (PV606531), and *N. spathiger* (PV606532). Phylogenetic analysis based on the ITS region (Fig. 5) showed clear separation between these species, each forming well-supported clades with bootstrap values > 90%, reflecting their genetic divergence and validating their morphological classification. The ITS sequences of all species were high-quality, showing ≥ 98–100% identity with reference sequences in GenBank, confirming the reliability of molecular identification.

**Fig. 6:**
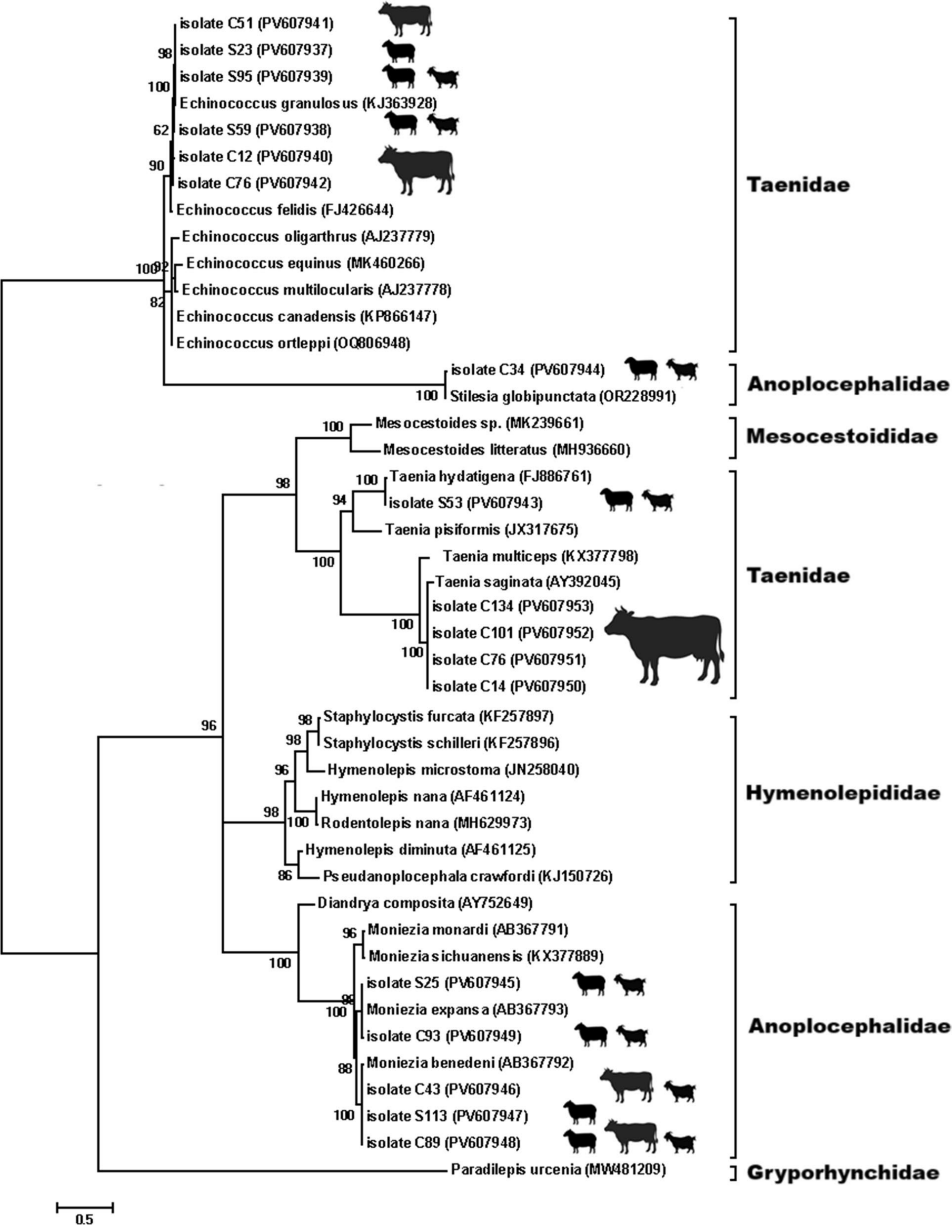
Evolutionary analysis by the Maximum Likelihood method of cestodes based on ITS region. The phylogeny was inferred using the Maximum Likelihood method and Hasegawa-Kishino-Yano (1985) model of nucleotide substitutions and the tree with the highest log likelihood (-30,141.96) is shown. The percentage of replicate trees in which the associated taxa clustered together (1000 replicates) is shown next to the branches

Eleven isolates of cestodes were sequenced, including six isolates of *E. granulosus* (PV607937–PV607942), four isolates of *T. saginata* (PV607950–PV607953), and one *T. hydatigena* (PV607943). Additionally, sequences of *M. expansa* (PV607945, PV607949), *M. benedeni* (PV607946–PV607948), and *S. globipunctata* (PV607944) were obtained. The phylogenetic tree (Fig. 6) revealed distinct clusters for each genus and species, with high intra-species similarity and strong bootstrap support. All *E. granulosus* sequences clustered with known G1/G3 strains, indicating that the dominant genotype in the region is the sheep strain.

**Fig. 7:**
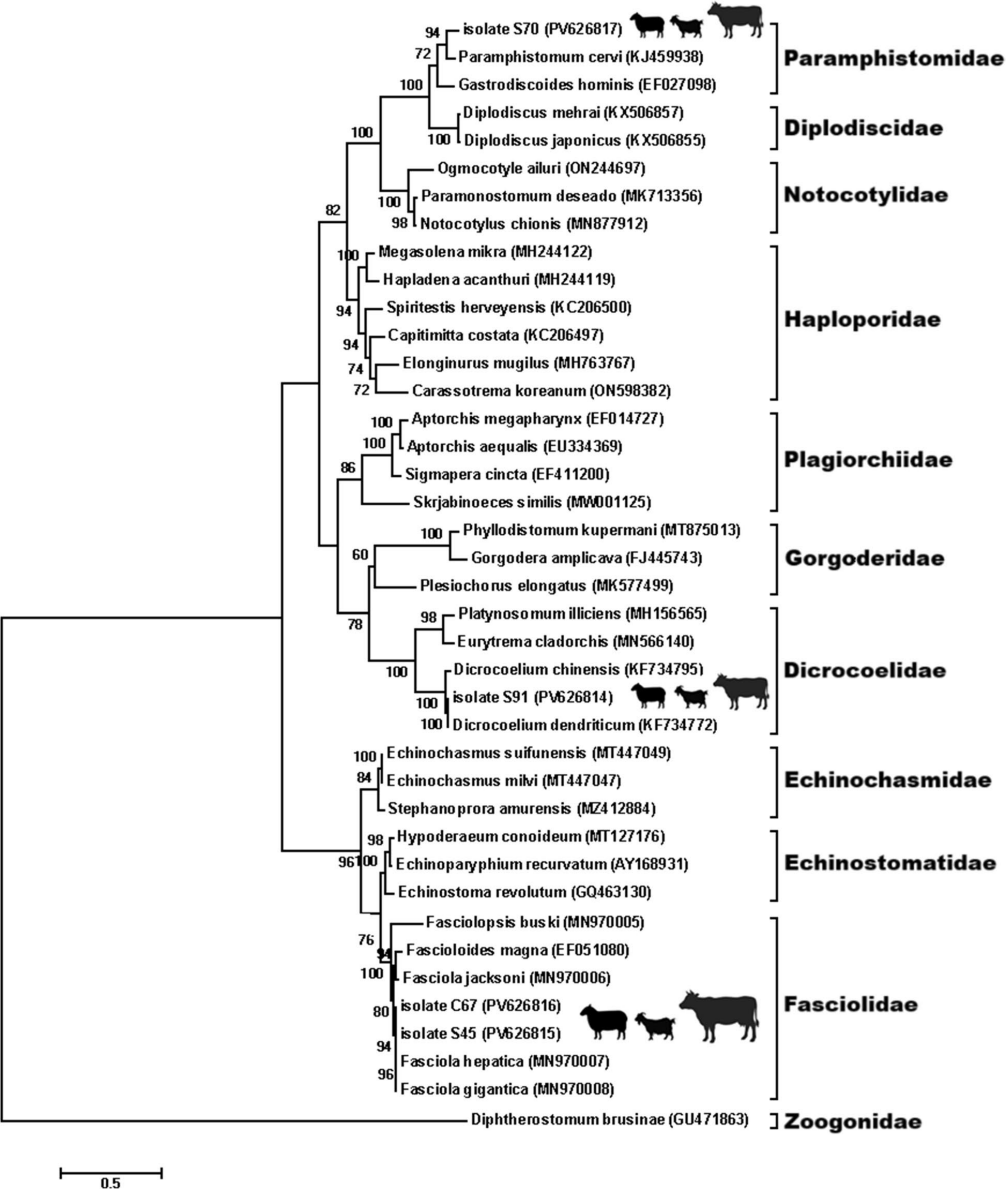
Evolutionary analysis by the Maximum Likelihood method of trematodes based on ITS region. The phylogeny was inferred using the Maximum Likelihood method and General Time Reversible model of nucleotide substitutions and the tree with the highest log likelihood (-35,389.24) is shown. The percentage of replicate trees in which the associated taxa clustered together (1000 replicates) is shown next to the branches

Four trematode species were identified and sequenced: *D. dendriticum* (PV626814), *F. hepatica* (PV626815), *F. gigantica* (PV626816), and *P. cervi* (PV626817). The phylogenetic tree (Fig. 7) clearly separated *F. hepatica* and *F. gigantica* into distinct clades, confirming their coexistence in the study area. The presence of *D. dendriticum* and *P. cervi* was also confirmed, with each forming monophyletic groups with closely related reference sequences from other regions of Iran and neighboring countries.

Remarkably, the ITS sequence of *L. serrata* (PV658352) was successfully amplified and submitted to GenBank, representing the first report of the ITS region for this species. Prior to this study, no ITS sequence of *L. serrata* had been available in public databases. This novel sequence provides a valuable genetic marker for future taxonomic and phylogenetic investigations of this zoonotic pentastomid.

## Discussion

This study provides a comprehensive assessment of the prevalence and distribution of helminthic infections in slaughtered livestock in Qazvin Province, Iran. The findings show a high parasitic load, particularly in sheep and goats, with zoonotic helminths showing a remarkable prevalence which has the potential to pose serious threats to food security and public health.

The overall prevalence of helminthic parasite infection in livestock examined in this study was 47.10%, which is relatively consistent with previous investigations in Iran including 56.6% in Qazvin [14], and 53.33% in Mazandaran [21], as well as other Asian countries in including Pakistan (58.59%) (T. [22]), Iraq (45.7%) [23], and Bangladesh (43.6%) [24].

Among the host species, sheep exhibited the highest incidence of infection, consistent with their grazing habits and greater exposure to parasitic contamination. Goats showed a moderate level of infection, while cattle had significantly lower rates. This disparity may be attributed to differences in management practices, grazing behaviour, and immune response. Sheep typically carry the heaviest worm burdens and therefore require more frequent anthelmintic treatment, followed by goats. In contrast, cattle tend to have lower parasite loads and develop more robust immunity over time [25]. Moreover, the relatively stable patterns in cattle may reflect housing practices or differences in grazing behaviour. This suggests species-specific differences in exposure and immunity that should be considered when designing targeted parasite control programs [26, 27].

Interestingly, no nematodes were detected in cattle in the present study. While this may accurately reflect the true infection status, several factors could contribute to this observation. Cattle in Qazvin Province are often raised under semi-intensive management systems with routine deworming programs, which may effectively reduce nematode burdens. Moreover, adult cattle tend to develop stronger acquired immunity against gastrointestinal nematodes compared to small ruminants, resulting in lower infection rates. Another possibility is that some nematode species, particularly those residing in the gastrointestinal tract, may have been present at low intensities below the detection limit of the post-mortem and coprological methods used. These factors together may explain the apparent absence of nematodes in cattle.

Our investigation revealed a diverse range of parasitic infections, including three species of nematodes, four species of trematodes, six species of cestodes (in both adult and larval stages), and a single species of pentastomid.

The most prevalent cestode identified across all host species was CE, which showed a notably high occurrence mirroring trends observed in previous studies on livestock populations in other parts of Iran [14, 28–30], and the neighbouring countries such as Iraq [31], Azerbaijan [32], and Turkey [33, 34], underscoring the endemic nature of this infection throughout the region.

A recent study in Qazvin Province showed that CE was the most prevalent infection, found in 310/1,440 sheep (21.5%) and in 115/810 goats (14.1%) [14]. It is projected that CE is accounted for 1 -3.6 million disability-adjusted life years (DALYs) worldwide, with the heaviest impact seen in low- and middle-income regions [35, 36]. In Qazvin Province, a decade-long survey documented 203 cases of CE, resulting in a surgical incidence rate of 1.49 per 100,000 people [37].

These findings are particularly concerning given the zoonotic potential of CE and its recognition as a significant public health concern in endemic areas.

In addition to CE, other zoonotic helminths identified in this study also pose notable risks to human health through distinct transmission routes [14, 38]. *Fasciola hepatica* can infect humans primarily through the consumption of raw or undercooked aquatic plants such as wild watercress or other leafy greens harbouring encysted metacercariae [39]. This route of infection is particularly relevant in rural areas of Iran where such plants are locally collected and consumed. Similarly, *L. serrata*, the causative agent of linguatulosis, may be transmitted to humans via the ingestion of raw or undercooked viscera (especially liver or lymph nodes) from infected herbivores, or through accidental ingestion of eggs shed by infected animals such as canids. Although human cases are rare, these practices underscore the need for public education on safe food handling and cooking, as well as strict meat inspection and control of stray dog populations in endemic areas [40].

Beyond human health risks, CE negatively impacts livestock productivity by reducing the quality and yield of outputs such as milk, meat, wool, and hides, and it is also associated with diminished reproductive performance [41]. Although slaughterhouses are officially overseen by the veterinary authorities, some municipal facilities lack proper infrastructure for the safe disposal of condemned organs. As a result, infected offal may become accessible to stray or domestic dogs, perpetuating the transmission cycle of cystic echinococcosis. Enhancing carcass inspection protocols and upgrading slaughterhouse facilities are critical steps in strengthening control efforts against the disease (A. [42]).

Among trematodes, *D. dendriticum* and *F. hepatica* were dominant. These zoonotic liver flukes are well-documented in Iranian livestock and are known to cause chronic infections that impair animal productivity [14, 29, 43]. The observed seasonal trends higher prevalence during spring and summer suggest environmental factors such as temperature and moisture significantly influence the life cycles of intermediate hosts (e.g., snails and ants), facilitating greater transmission during warmer and wet months [44–46].

Gastrointestinal nematode infections contribute to serious health concerns in livestock and widespread economic impacts worldwide. Sheep that graze on contaminated pastures are vulnerable to infection by the infective third-stage larvae of these parasites [47]. Among gastrointestinal nematodes, *N. spathiger* was the most prevalent, followed by the zoonotic species including *M. marshalli* and *H. contortus*, primarily affecting small ruminants. These parasites contribute to gastrointestinal distress and poor weight gain, with potential economic implications for farmers [2, 48]. The primary method for controlling livestock parasitic nematodes involves anthelmintic medications. Even with strategic and well-timed treatments, control measures are costly and often not fully effective. Furthermore, the frequent and excessive use of anthelmintics has contributed to widespread resistance issues within nematode populations. Therefore, the need for more effective control methods against parasitic nematodes is evident and has garnered significant worldwide attention [2, 49].

Furthermore, bovine cysticercosis is considered a major food safety issue due to its role in transmitting human taeniasis. It also poses a serious economic burden, as infected carcasses must be condemned, frozen, or downgraded. The disease is caused by *C. bovis*, the larval form of *Taenia saginata*, which resides in the human small intestine [50].

The integration of molecular analysis in this study not only confirmed the morphological identification of helminths but also provided more robust species-level resolution, especially for morphologically similar or cryptic taxa. The ITS region of the ribosomal DNA, used in this study, is widely recognized for its discriminatory power among helminths, particularly within nematodes, cestodes, and trematodes [18, 19].

Phylogenetic analysis using MEGA11 revealed clear monophyletic clustering of *H. contortus*, *M. marshalli*, and *N. spathiger*, supporting their species boundaries and evolutionary divergence. The placement of *N. spathiger* in a distinct clade aligns with previous molecular surveys in small ruminants in the region and highlights its consistent presence in Iran’s parasitic fauna [18].

Among cestodes, the multiple isolates of *E. granulosus* formed a tight cluster, confirming intra-specific homogeneity across different hosts and supporting their role as the predominant agent of cystic echinococcosis in the region. The presence of G1 and G3 genotypes was previously documented in livestock in Qazvin Province using mitochondrial COX1 genes [51].

The presence of several *T. saginata* isolates with high sequence similarity also suggests sustained transmission in cattle, while the identification of both *M. expansa* and *M. benedeni* in sheep and goats aligns with observed seasonal patterns and emphasizes the value of molecular methods in differentiating closely related species [52, 53].

In the trematode group, the sequences of *F. hepatica* and *F. gigantica* clustered into separate lineages, reaffirming the sympatric distribution of these two species in Iran. This sympatry could have implications for hybridization or misdiagnosis in regions where both species coexist. Furthermore, the identification of *P. cervi* through ITS analysis provided a reliable confirmation of this rumen fluke, often overlooked in traditional diagnosis due to its low pathogenicity and ambiguous morphology [54].

The most notable molecular achievement in this study was the first successful amplification and submission of the ITS region of *L. serrata* to GenBank (PV658352). To our knowledge, this is the first ITS sequence reported for this species globally. While previous molecular studies on *L. serrata* have focused primarily on mitochondrial genes such as COX1, the ITS region remained uncharacterized [55, 56].

Given the zoonotic importance of *L. serrata*, particularly in endemic regions like Iran, the availability of ITS data offers a new molecular tool for accurate identification, population genetics, and phylogenetic studies. This novel sequence also provides a reference for future investigations into genetic diversity and host specificity of *L. serrata*, and fills a significant gap in the molecular taxonomy of pentastomids [57].

This finding underscores the importance of combining morphological and molecular approaches in parasitological research, not only for confirmation but also for the discovery and documentation of previously uncharacterized genetic markers [19].

All molecularly confirmed species in this study were registered in GenBank (accession numbers PV606530–PV658352), contributing valuable reference sequences for future research and aiding regional helminth surveillance. The availability of such genetic data enhances the reliability of species identification in epidemiological studies and facilitates the detection of emerging strains or potential anthelmintic resistance patterns.

Seasonal and environmental fluctuations, including climate change, play a key role in the emergence and spread of infectious diseases. The temporal patterns observed in our study underscore the strong impact of seasonal and environmental factors on parasite transmission, particularly in small ruminants. Accordingly, the seasonal distribution patterns revealed spring as the peak period for transmission, most likely due to environmental conditions such as moderate temperatures and increased humidity that promote the survival and activity of intermediate hosts. These favourable climatic factors may enhance egg viability and increase host–parasite contact rates, contributing to the heightened transmission observed during this season [58].

Interestingly, the seasonal pattern of *L. serrata* infection in cattle showed an unusual bimodal trend, with peaks in both spring and winter. This fluctuation may be associated with ecological and management factors influencing the life cycle of this parasite. *L. serrata* relies on canids as definitive hosts, and seasonal variations in contact between cattle and dogs, particularly stray or shepherd dogs around slaughter facilities and pastures, may contribute to these patterns. Increased exposure during spring could result from greater outdoor grazing activity and environmental contamination, whereas winter peaks may reflect longer larval survival in cooler conditions or delayed detection following earlier infections. Alternatively, this trend might indicate intermittent transmission linked to feeding or slaughtering practices. Further longitudinal and molecular investigations are warranted to clarify the dynamics of *L. serrata* infection across seasons.

These findings collectively support the implementation of seasonal parasite control programs targeting high-risk periods in spring and summer to reduce parasite burden and limit transmission.

The primary strategies for controlling helminthic parasites involve prophylactic anthelmintic treatment alongside effective grazing management. Given the high prevalence in sheep and the distinct seasonal peak in spring, a targeted pre-spring anthelmintic treatment program for small ruminants could be a highly effective and resource-efficient strategy for the region. Key control measures include strategic grazing practices, the development of natural or vaccine-induced immunity, biological control methods, and the responsible use of anthelmintics [59]. Good husbandry practices such as managing stocking density, implementing rotational grazing, and maintaining hygienic pastures can serve as alternative means of parasite control [60]. The most effective way to prevent gastrointestinal helminth infections is to minimize animal exposure to contaminated environments. However, complete separation of livestock from areas harbouring intermediate hosts is only practical in intensive farming systems, which remain limited in implementation across the country. In contrast, under the traditional communal grazing systems common in Ethiopia, where animals share grazing areas, measures like rotational grazing or provision of parasite-free pastures are often impractical [61].

## Conclusion

The observed burden of helminth infections particularly zoonotic parasites in livestock from Qazvin Province underlines the necessity of coordinated and effective control strategies. These parasites not only compromise animal health and productivity but also pose serious threats to food safety and public health.

The seasonal dynamic underscore the importance of timely and season-specific control strategies including pre-spring deworming and pasture rotation. Priority should be given to small ruminants, particularly sheep, which harboured the highest parasite loads and contribute substantially to environmental contamination. Future studies should further focus on molecular characterization of the helminths, assessment of anthelmintic resistance, and evaluation of human exposure to zoonotic species through dietary and occupational routes. Access to detailed regional data on parasite species, prevalence, and transmission patterns remains essential for developing effective and sustainable control programs.

Taken together, molecular characterization significantly strengthened the diagnostic resolution of this study, allowed confirmation of zoonotic species, and highlighted the high diversity of helminths affecting livestock in Qazvin Province. The integration of morphological and molecular tools is thus essential for accurate diagnosis, effective surveillance, and informed control strategies in both veterinary and public health contexts. These findings support a One Health approach that integrates veterinary, environmental, and public health sectors.

## Data Availability

The data generated during and analyzed during the study are available from the corresponding author on reasonable request.
